# EPLIN Expression in Gastric Cancer and Impact on Prognosis and Chemoresistance

**DOI:** 10.3390/biom11040547

**Published:** 2021-04-08

**Authors:** Wenjing Gong, Jianyuan Zeng, Jiafu Ji, Yongning Jia, Shuqin Jia, Andrew J. Sanders, Wen G. Jiang

**Affiliations:** 1 Department of Oncology, Yantai Yuhuangding Hospital, Medical College, Qingdao University, Yantai 264000, China; gongwenjingyt@163.com; 2 Cardiff China Medical Research Collaborative (CCMRC), Division of Cancer and Genetics (DCG), Cardiff University School of Medicine, Cardiff CF14 4XN, UK; ZengJ8@cardiff.ac.uk; 3 Key Laboratory of Carcinogenesis and Translational Research (Ministry of Education), Department of Gastrointestinal Surgery, Peking University Hospital and Institute, No. 52 Fucheng Road, Haidian District, Beijing 100142, China; jiafuji_pkuos@sina.com (J.J.); yongningjia@bjmu.edu.cn (Y.J.); jiashuqin2014@163.com (S.J.)

**Keywords:** EPLIN, gastric cancer, clinicopathology, prognosis, chemoresistance

## Abstract

Epithelial protein lost in neoplasm (EPLIN) has been implicated as a suppressor of cancer progression. The current study explored EPLIN expression in clinical gastric cancer and its association with chemotherapy resistance. EPLIN transcript expression, in conjunction with patient clinicopathological information and responsiveness to neoadjuvant chemotherapy (NAC), was explored in two gastric cancer cohorts collected from the Beijing Cancer Hospital. Kaplan-Meier survival analysis was undertaken to explore EPLIN association with patient survival. Reduced EPLIN expression was associated with significant or near significant reductions of overall, disease-free, first progression or post-progression survival in the larger host cohort and Kaplan Meier plotter datasets. In the larger cohort EPLIN expression was significantly higher in the combined T1 + T2 gastric cancer group compared to the T3 + T4 group and identified to be an independent prognostic factor of disease-free survival and overall survival by multivariate analysis. In the smaller, NAC cohort, EPLIN expression was found to be significantly lower in tumour tissues than in paratumour tissues. EPLIN expression was significantly associated with responsiveness to chemotherapy which contributes to overall survival. Together, EPLIN appears to be a prognostic factor and may be associated with patient sensitivity to NAC.

## 1. Introduction

Gastric cancer is a common worldwide cancer which is diagnosed in excess of 1 million people every year according to the International Agency for Research on Cancer (IARC) GLOBOCAN project [1]. Despite its incidence and mortality decreasing over the past five years, it remains the third most common cause of cancer death [1]. The main failures of cancer treatment are local recurrence and metastasis. Additionally, the acquisition of chemotherapy resistance is also a common reason contributing to treatment failure [2]. Recent systematic review and meta-analysis of the safety and efficacy of third and later line therapy, following failure of initial therapy, in patients with advanced or metastatic gastric or gastroesophageal cancer demonstrated their potential benefit and highlighted the need for focused research on improving patient selection in order to identify those who could benefit from such therapies [3]. Greater understanding of the mechanisms involved in these processes is essential to improve patient management and treatments. For example, studies focused on the role of claudin-18 in mouse models has highlighted that loss of claudin-18 can drive neoplastic development in the mouse stomach [4]. Research aimed at identifying such drivers of progression or those involved in therapy response is vital in aiding future patient management and treatment regimes.

Epithelial protein lost in neoplasm (EPLIN) is an actin binding cytoskeletal protein involved in tumour progression, acting as a suppressor of cancer cell growth, invasion and migration, and is frequently lost in numerous cancer types and cell lines [5,6,7,8,9]. EPLIN, also known as LIMA1 (LIM domain and actin binding-1), was first discovered due to its alterations between normal oral epithelial and human papilloma virus (HPV) immortalised epithelial cells [10]. The EPLIN gene is located at chromosome 12q13.12 and has two isoforms; a longer EPLIN-β and a shorter EPLIN-α isoform and has been found to be expressed in various tissues [9,11,12]. EPLIN has been demonstrated to contribute to stabilisation of the actin cytoskeleton and regulation of cell dynamics by interacting with filamentous actin (F-actin), illustrating its crucial role in cell motility [13,14,15]. Additionally, EPLIN engages in cell-cell adherence junctions by linking the cadherin-β-catenin-α-catenin-EPLIN-F-actin complex. It is also suggested to interact with paxillin [11,16,17,18] and plays an indispensable role in stabilising apical-basal polarity in epithelial cells [9]. Previous studies revealed that EPLIN negatively correlates with epithelial mesenchymal transition (EMT), invasiveness, metastasis, poor prognosis, mortality and therapeutic resistance in human tumours such as prostate, breast, ovarian and oesophageal cancers [6,7,9,19,20], and that epidermal growth factor (EGF) could cause protein phosphorylation and turnover of EPLIN through an extracellular signal-regulated kinase (ERK) signal pathway to influence EMT [21].

Collectively, EPLIN is known to contribute as a tumour/metastasis suppressor affecting actin dynamics, cytoskeletal organisation, motility and cancer progression [9,20]. Loss of EPLIN may account for dysregulation of cytoskeletal dynamics, alterations of cell motility and cell-cell adhesion disruption which is believed to promote tumour proliferation, invasion and migration [14,15]. Recently, scientific interest in EPLIN has grown, however its relevance to gastric cancer remains unknown. In this study, we investigated the expression of EPLIN in two gastric cancer cohorts to explore its correlation with clinicopathological factors and its importance in responsiveness to neoadjuvant chemotherapy (NAC).

## 2. Materials and Methods

### 2.1. Gastric Cancer Tissue Collection 

Two gastric cohorts were utilised in the current study. The first cohort contained 320 gastric cancer tissue samples and 175 paired normal background tissue samples, and the second cohort contained 78 gastric cancer tissue samples and 80 normal background tissue samples, available from a cohort comprising cancer and normal matched patient tissues, and had additional patient information regarding patient responsiveness to NAC. Both cohorts were obtained from the Beijing Cancer Hospital and have been previously reported [22,23]. Tissue samples were immediately collected in labelled universal containers after surgery and stored in liquid nitrogen until used. The process was checked by a consultant pathologist. This collection was supported by local ethics committee (Peking University Cancer Hospital Research Ethics Committee, ethics number 2006021) with patients’ consent. Patients were routinely followed up and their clinical pathological details were obtained. Clinical information of the patients and patients with NAC is outlined in Table 1 and Table 2, respectively.

### 2.2. Tissue Processing, RNA Extraction and cDNA Generation

Tissue samples were homogenised in TRI reagent (Sigma-Aldrich, Dorset, UK) using a handheld homogeniser (Cole Parmer, Cambridgeshire, UK) and RNA extraction undertaken in accordance with the manufacturers’ guidelines. RNA concentrations were standardised, to allow sample normalisation, and used as a template to generate cDNA using a GoScript reverse transcription mix, Oligo (dT) kit (Promega, Southampton, UK) in accordance with the manufacturers’ guidelines.

### 2.3. Real-Time Quantitative PCR (qPCR)

EPLIN transcript expression was quantified within the tissue cohorts using an Amplifluor^TM^ Uniprimer^TM^ Universal qPCR system (Intergen Inc., Oxford, UK). Forward and reverse primers, containing a Z sequence (5′-ACTGAACCTGACCGTACA-3′) required for the incorporation of the FAM-tagged Uniprimer^TM^ probe and fluorescent detection, were as follows; EPLIN forward AAGCAAAAATGAAAACGAAG and Z tagged reverse ACTGAACCTGACCGTACAGACACCCACCTAGCAATAG. Each reaction contained forward primer, reverse primer, tissue sample cDNA, Uniprimer^TM^ and 2× Precision FAST qPCR master mix (Primer Design, Eastleigh, UK) and was carried out using a StepOnePlus^TM^ Real-Time PCR System (Thermo Fisher Scientific, Leicestershire, UK) under the following conditions: 95 °C for 10 min, 100 cycles of 95 °C for 10 sec, 55 °C for 35 sec and 72 °C for 10 sec. The transcripts were quantified alongside an internal standard, containing known transcript copy numbers of a reference gene. Standard samples, serial diluted from 10^8^ to 10^1^ concentrations were run alongside unknown samples on the same plates, using the same reaction setup. From this a standard curve was generated and used to subsequently determine relative transcript copy numbers of the unknown samples.

### 2.4. Kaplan-Meier Plotter Gastric Cancer Database

The association of EPLIN expression with survival outcomes was also analysed using the publicly available Kaplan–Meier Plotter gastric cancer database [24]. The following criteria were selected for the analysis. Affymetrix ID 217892_s_at was used to analyse median survivals of first progression (FP), overall survival (OS) and post progression survival (PPS) comparing high LIMA1 expression to low LIMA1 expression based on cut off levels (median).

### 2.5. Statistical Analysis

Mann–Whitney U tests or Kruskal–Wallis tests were used to compare expression between patient group and were undertaken using Minitab (version 14) (Minitab Ltd., Coventry, UK) and SigmaPlot (Version 11) (Systat Software Inc., San Jose, CA, USA) statistical software. Kaplan-Meier survival analysis and Cox hazardous proportion analysis were undertaken using the SPSS statistical software (version 11; SPSS, Chicago, IL, USA). Box plots were prepared using GraphPad Prism software (version 8). A *p* < 0.05 was regarded as statistically significant.

## 3. Results

### 3.1. Transcript Expression of EPLIN in Clinical Gastric Cancer and Association with Clinicopathological Information

EPLIN expression within the first gastric cancer cohort was analysed according to gender, the depth of primary tumour infiltration into the gastric wall (T category), nodal status, metastasis status, TNM stage, Borrmann classification, histopathologic type, differentiation and survival status (Table 1).

**Figure 1:**
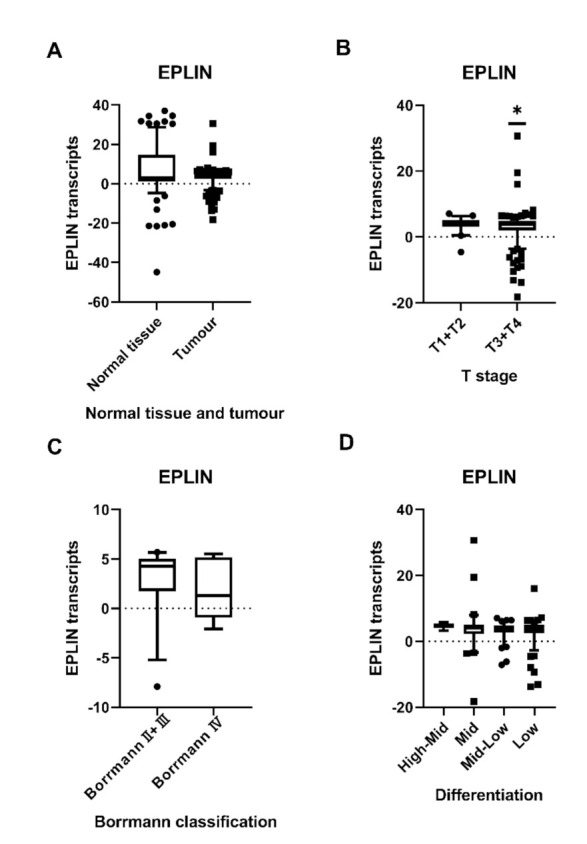
Association between EPLIN expression and T stage, Borrmann classification and differentiation in gastric cancer. (**A**): No difference was noted in EPLIN expression between tumour tissues and normal tissues. (**B**): The combined T1 + T2 group had significantly higher EPLIN expression than combined T3 + T4 group. (**C**): The combined BorrmannⅡ + BorrmannⅢ group had higher EPLIN transcript expression compared to BorrmannⅣ group. (**D**): There was a decline in EPLIN expression from high-middle differentiation to low differentiation. High differentiation group was not shown because of low sample number. Shown as median log_10_ values with 95% cl. * *p* < 0.05.

No significant difference in EPLIN transcript expression was found between gastric tumour tissues and normal tissues (*p* = 0.7288) (Figure 1A). Interestingly, EPLIN expression appeared to have a negative correlation with the depth of tumour infiltration into the stomach wall and it was noted that the combined T1 + T2 group, combined to give a broader overview and to combat low sample numbers in individual groups, had significantly higher levels of EPLIN transcript compared to the more invasive combined T3+T4 group (*p* = 0.0421) (Figure 1B), though no-significant differences were observed between the individual groups (Table 1). Additionally, the combined Borrmann Ⅱ + Borrmann Ⅲ group displayed higher, though non-significantly, levels of EPLIN expression compared with Borrmann Ⅳ group (Figure 1C) and no significant differences noted between individual groups (Table 1). Furthermore, EPLIN transcript levels tended to be higher in the more highly differentiated tissues compared to those with lower levels of differentiation, though statistical significance was not reached (Figure 1D).

No significant difference was noted between EPLIN expression and gender, node status, TNM stage, metastasis status, histopathologic type and survival status (Table 1).

### 3.2. Relationship Between EPLIN Expression and Overall Survival (OS) in Patients with Gastric Cancer

We also compared EPLIN expression in conjunction with clinical outcomes. Data was available for 307 patients followed up for 120 months. Patients were divided into high EPLIN and low EPLIN expression groups, based on cut off level (median) and compared to patient survival rates.

**Figure 2:**
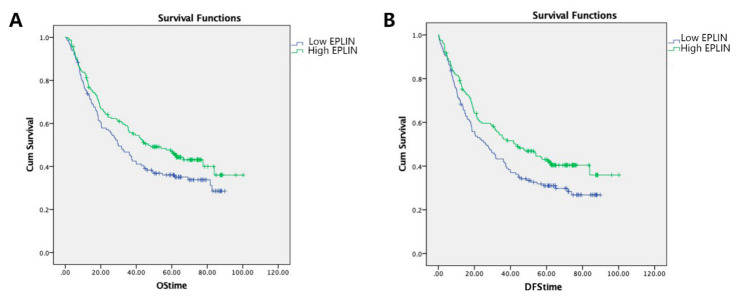
Relationship between EPLIN expression and clinical survival outcomes in gastric cancer cohort. (**A**): Longer overall survival was seen in patients with higher EPLIN expression compared to those with relatively lower EPLIN expression. (**B**): Patients with higher levels of EPLIN transcript had a significantly longer disease-free survival than those with lower levels of EPLIN transcript.

Patients with high EPLIN expression had a longer overall survival (median = 46.8 months, 95% cl: 28.893–64.707 months) compared to those with low EPLIN expression (median = 29.9 months, 95% cl: 21.361–38.439 months) and this was found to be close to statistically significant (*p* = 0.067) (Figure 2A). Moreover, multivariate analysis identified EPLIN (*p* = 0.024), TNM stage (*p* < 0.001), T stage (*p* < 0.001), nodal involvement (*p* < 0.001), metastasis status (*p* < 0.001), invasion (*p* < 0.001) and embolism (*p* < 0.001) as independent prognostic factors for overall survival, with univariate analysis also identifying EPLIN as a prognostic indicator of survival with close statistical significance (Table 3).

### 3.3. Association of EPLIN Expression and Disease-Free Survival (DFS) in Patients with Gastric Cancer

Similarly, EPLIN expression levels were compared to patient Disease-Free Survival (DFS). Patients with high levels of EPLIN transcript displayed significantly improved DFS compared to those with low levels (high EPLIN expression median = 42.667 months, 95% cl: 27.170–58.183 months; low EPLIN expression median = 26.433 months, 95% cl: 17.466–35.401 months; *p* = 0.025) (Figure 2B). Furthermore, multivariate analysis found that EPLIN (*p* = 0.015), TNM stage (*p* < 0.001), T stage (*p* < 0.001), nodal involvement (*p* < 0.001), metastasis status (*p* < 0.001), invasion (*p* < 0.001) and embolism (*p* < 0.001) were seven independent prognostic factors for DFS which was consistent with our EPLIN univariate analysis (Table 3).

### 3.4. Association of EPLIN/LIMA1 Expression with Gastric Cancer Patient Survival in Publicly Available Databases

We analysed the relationship between the expression of LIMA1 and the OS, FP and PPS of gastric cancer patients. OS (*n* = 875), FP (*n* = 640) and PPS (*n* = 498) curves were generated and obtained from the public online database, Kaplan-Meier plotter, which includes gene expression and survival information of gastric cancer patients [24]. This resource was used to explore LIMA1 (Affymetrix ID: 217892_s_at) expression and its relation to gastric cancer patient survival rates.

**Figure 3:**
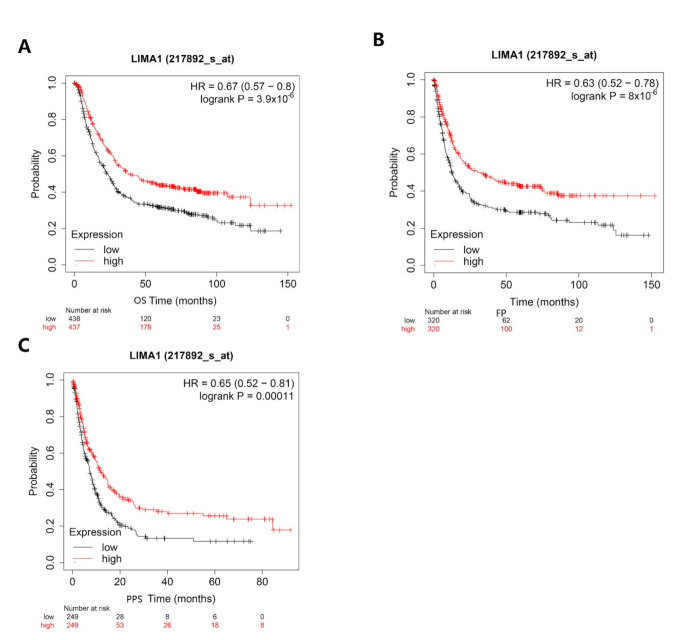
Analysis of LIMA1/EPLIN association with gastric cancer survival in online database. (**A**): Patients with high LIMA1 expression had a significantly longer overall survival (OS) than those with low LIMA1 expression. (**B**): Patients with high LIMA1 expression had a significantly longer first progression (FP) survival time than those with low LIMA1 expression. (**C**): Patients with high LIMA1 expression had a significantly prolonged PPS compared to those with low LIMA1 expression. Images produced and obtained using Kaplan-Meier Plotter website [24].

Gastric cancer patients with high expression of LIMA1 had a significantly longer OS than those with low expression of LIMA1 (HR = 0.67, 95% cl 0.57–0.8; *p* = 3.9 × 10^−6^) (Figure 3A). Similarly, high expression levels of LIMA1 were also significantly associated with increased FP time in gastric cancer patients (HR = 0.63, 95% cl 0.52–0.78; *p* = 8 × 10^−6^) (Figure 3B). Significantly longer PPS was also observed in gastric cancer patients who had high LIMA1 expression compared with those with relatively low LIMA1 expression (HR = 0.65, 95% cl 0.52–0.81; *p* = 0.00011) (Figure 3C).

### 3.5. Implication of EPLIN Transcript Expression in Neoadjuvant Chemotherapy (NAC)

**Figure 4:**
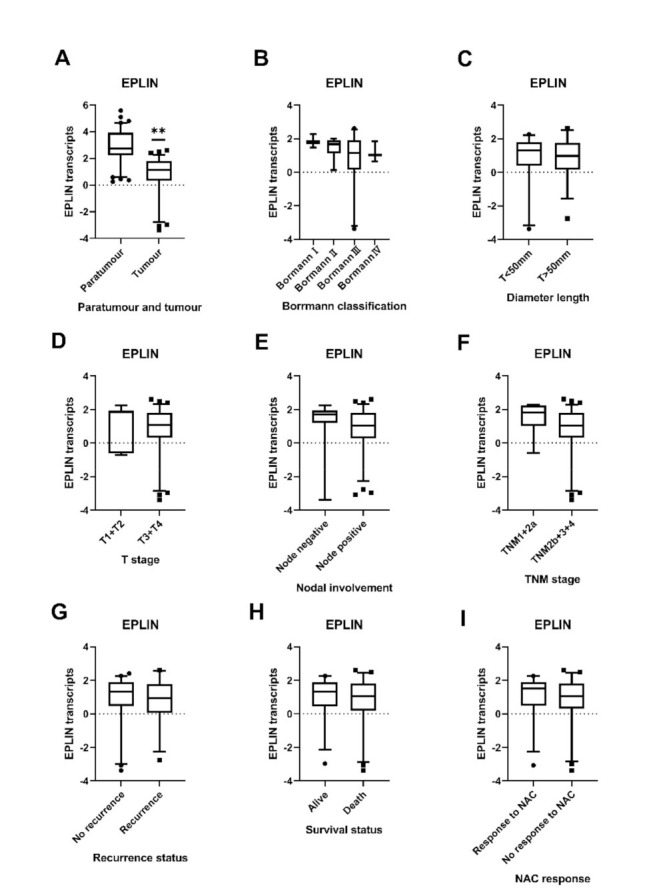
Correlation between EPLIN transcripts and clinicopathology information in gastric cancer with NAC. (**A**) EPLIN expression was significantly decreased in tumour tissues compared to paratumour tissues. (**B**) A downward trend of EPLIN expression was observed from BorrmannⅠ to BorrmannⅣ. (**C**) Tumours with diameter length > 50 mm had a lower EPLIN expression compared to that with diameter length < 50 mm group. (**D**): EPLIN expression was expressed at a higher level in the combined T1 + T2 group compared to combined T3 + T4 group. (**E**): EPLIN expression was increased in the node negative group compared to that in node positive group. (**F**): EPLIN expression with earlier stage TNM1+2a was higher than those with advanced stage TNM2b+3+4. (**G**): EPLIN expression was higher in no recurrence group compared to those in recurrence group. (**H**): EPLIN expression in the alive survival group was increased compared to those in the death group. (**I**): Higher levels of EPLIN expression was noted in tumours with response to NAC group than those with no response to NAC group. Shown as median log_10_ values with 95% cl. ** *p* < 0.001.

A second smaller cohort containing clinical information of gastric cancer patients treated with NAC (*n* = 78) and normal tissues (*n* = 80) was utilised to explore the implications of EPLIN expression in patients with NAC, demonstrating the association between EPLIN expression and the clinical pathologic features among these patients. Similar to the previous cohort, the expression of EPLIN was analysed in conjunction with available clinicopathological information (Table 2). Interestingly, EPLIN transcript expression was found to be associated with tumorigenesis in patients with NAC, with significant reductions observed in tumour tissues compared with paratumour tissues (*p* < 0.001) (Figure 4A). A downward trend in EPLIN transcript expression was observed from BorrmannⅠ to BorrmannⅣ but this was not statistically significant (Figure 4B). Combined analysis, designed to give a broader overview and combat low sample numbers in such groups and looking at EPLIN expression in BorrmannI + BorrmannII groups compared to BorrmannIII + BorrmannIV groups demonstrated a generally higher level of EPLIN expression in the combined BorrmannI + BorrmannII stage tumours compared to the combined BorrmannⅢ + BorrmannⅣ stage tumours groups, with near significance observed (*p* = 0.071). Additionally, tumours with diameter length >50mm had a non-significantly lower EPLIN expression compared to that with diameter length <50mm (Figure 4C) and elevated, though non-significantly, EPLIN expression was observed in the combined T1 + T2 group compared to combined T3 + T4 group with deeper tumour invasion (Figure 4D). Furthermore, EPLIN transcript levels were observed to be non-significantly increased in patients who had no lymph node metastasis compared to those who had lymph node metastasis (*p* = 0.0979) (Figure 4E). Similarly, increased EPLIN transcript level was noted in earlier stage TNM1+2a tumour tissues than that with advanced stage TNM2b+3+4, though this was not significant (Figure 4F). Moreover, patients accepting NAC were divided into recurrence and no recurrence groups. EPLIN transcript levels were found to be higher in the no recurrence group compared to those in recurrence group, though this was not found to be statistically significant (Figure 4G). Additionally, EPLIN expression in patients who were alive was higher than those who died but no significant difference was observed (Figure 4H).

### 3.6. Relationship between EPLIN Transcript Expression and Prognosis in Patients with NAC and Chemosensitivity

We next explored the potential implications between EPLIN transcript expression and responsiveness to chemotherapy, comparing EPLIN transcript expression between groups of patients who had or had not responded to NAC. A non-significantly higher level of EPLIN transcript was observed in tumours responsive to NAC than those with no response to NAC (Figure 4I).

**Figure 5:**
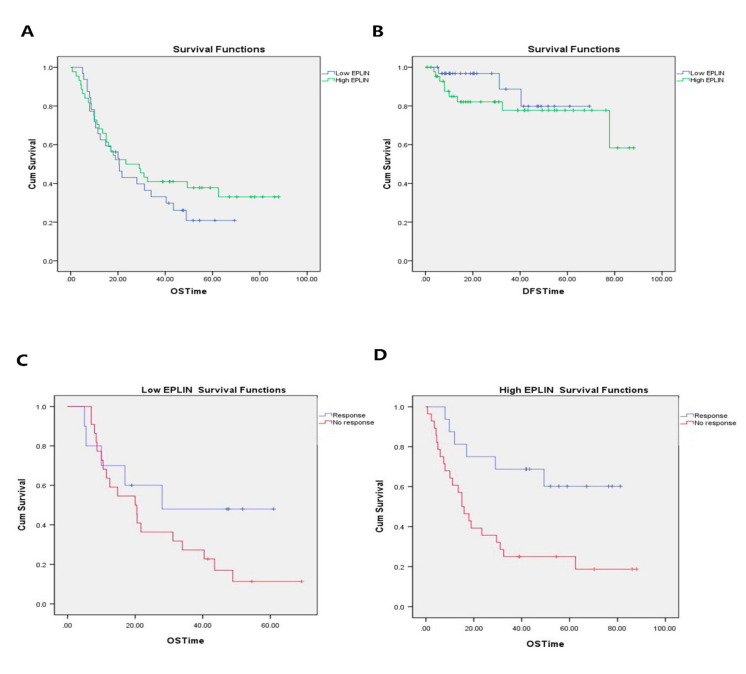
Relationship between EPLIN transcript expression and prognosis in patients with NAC and chemoresistance. (**A**): Patients with NAC expressing higher EPLIN levels seemed to have a slightly longer OS than those expressing lower EPLIN levels. (**B**): Patients with low EPLIN expression had better rates of disease-free survival in the early time period than those with high EPLIN expression, but in the later time period the rates of disease free in both groups were similar. (**C**): In patients with low EPLIN expression, the overall survival distribution was undertaken based on the different levels of responsiveness to NAC. (**D**): In patients with high EPLIN expression, the overall survival distribution was conducted for the different levels of tumour response to NAC.

To investigate the effect of EPLIN on the prognosis of gastric cancer patients with NAC, 76 patients with NAC were followed up for 100 months and OS and DFS analysed between groups of patients with high or low EPLIN expression levels. Longer OS rates were seen in patients with high EPLIN expression (median = 23.367 months; 95% cl: 8.199–38.535 months) compared to those with low EPLIN expression (median = 20.5 months; 95% cl: 14.306–26.694 months), but it did not reach statistical significance (Figure 5A). Patient DFS was also examined to further explore the role of EPLIN in prognosis (Figure 5B). EPLIN expression levels did not have any significant effects on disease free survival, though interestingly, particularly in the initial stages, the lower EPLIN expression group appeared to have better DFS rates.

Regarding the EPLIN association with chemosensitivity, we plotted OS curves for patients with different levels of responsiveness to NAC. In the patient group with low EPLIN expression, the mean OS in tumours responsive to NAC was 36.39 months (95% cl: 20.941–51.839) and that in tumours with no response to NAC was 26.166 months (95% cl: 17.726–34.606 months) (Figure 5C). Similarly, OS in patients with high EPLIN expression was also conducted based on levels of responsiveness to NAC. It was noted that the mean OS in tumours responsive to NAC was 57.821 months (95% cl: 42.716–72.926 months) and that in tumours with no response to NAC was 30.247 months (95% cl: 18.539–41.956 months) (Figure 5D). It was noted that the survival distribution for different levels of NAC response in high EPLIN group was wider than that in the low EPLIN group (*p* = 0.004). It suggested that EPLIN expression significantly influenced tumour responsiveness to NAC which led to overall survival distribution.

## 4. Discussion

Gastric cancer is one of the most aggressive cancers in the world with a high mortality rate. Despite improvements, gastric cancer still has poor prognosis due to low rates of early diagnosis, limited treatments and tumour heterogeneity [25]. Recurrence, metastasis and drug resistance are the main causes leading to treatment failure [2].

EPLIN is widely expressed in normal epithelial cells but frequently lost in cancer cells and tissues [6,7,8,18,19].

However, the role of EPLIN in gastric cancer progression remains largely unknown. Our present study explores the significance of EPLIN expression in clinical cohorts of gastric cancer, its usefulness as a prognostic factor and its potential relationship with responsiveness to NAC. The initial, larger cohort of gastric cancer was analysed to assess the association between EPLIN expression and clinicopathological details and prognosis, with the second, smaller cohort containing patients receiving NAC, assessed to explore the role of EPLIN in responsiveness to chemotherapy. There are some differences in the observed correlations and trends between our two cohorts. In this study, we found gastric cancer patients with low EPLIN expression were more likely to have cancer infiltration to the gastric wall and reduced tumour differentiation. In addition, the present study assessed the role of EPLIN in the prognosis of gastric cancer. Our study firstly established that high transcript levels of EPLIN were notably associated with improved DFS in gastric cancer and that increased EPLIN expression was also related to a long OS with near significance. In the meantime, we used Kaplan–Meier Plotter database to analyse the prognostic value of EPLIN in larger publicly available datasets, the outcome of which was consistent with our present study and indicated a significant relationship in these larger cohorts. Multivariate analysis also identified EPLIN as an independent prognostic factor of DFS and OS in gastric cancer. However, EPLIN expression had no effect on T stage, node status, metastasis status, TNM stage, Borrmann classification and histopathology. There are some limitations about the relationship between EPLIN expression and clinicopathology and prognosis in our research because of the limited number of cases in some subtypes which may limit statistical significance. Collectively, the most significantly important observation in our present study is the relationship between EPLIN and clinical outcome of gastric cancer suggesting that EPLIN may serve as a positive prognosis factor and may also inhibit tumour depth invasion and promote differentiation.

This clinical observation may be explained at the cellular and molecular level. Actin-binding proteins are essential in regulating the dynamics of actin bundles and branched actin filaments in epithelial cells which are involved in dynamic stabilisation, cell matrix adhesion and cell migration [26]. EPLIN has been identified as an actin-binding protein with EPLIN-α and EPLIN-β isoforms. Taha et al. suggested that EPLIN-α targeted to control membrane protrusion dynamics while EPLIN-β aims to stabilise stress fibres [26]. Previous studies demonstrated that EPLIN functions as a connection between the E-Cadherin-β-Catenin-α-Catenin complex and actin filaments to maintain epithelial phenotypes, stabilise actin cytoskeletal networks and maintain functional epithelial junctions [20]. EGF phosphorylates EPLIN which leads to EPLIN degradation via an ERK1/2 signalling pathway [9]. EPLIN phosphorylation or deletion may cause instability of the circumferential actin bundle which weakens the cell-cell contacts resulting in loss of cell-cell adhesion and early events of EMT [27]. EPLIN has also been demonstrated to modulate EMT via p53 [28]. In keeping with this, EPLIN has been strongly linked to regulating the processes of cellular invasion and motility [14,18,19,29]. Jiang et al. observed there was a correlation between low EPLIN transcripts and clinical outcomes in breast cancer, with reduced EPLIN transcript expression observed in tumour compared to normal tissues, in grade 2 and grade 3 cancers compared to grade 1 cancer and in TNM4 stage tumours compared to TNM1 stage tumours and low EPLIN transcript expression associated with poorer patient outlooks and reduced overall and disease-free survival [7]. EPLINα overexpression in MDA-MB-231 cells could reduce tumour growth and migration [7]. In oesophageal cancer, the expression of EPLIN was lower in aggressive tumours such as later TNM stage tumours, tumours with deep infiltration, node-positive tumours and tumours with low levels of differentiation. Enhanced EPLINα expression in the human oesophageal cancer cell line KYSE150 led to reduced cell invasion and decreased cell growth, but had no influence on cellular motility [8]. EPLIN was also inversely associated with the aggressiveness and outcome of ovarian cancer [19] and was similarly seen to be reduced in tumour compared to normal pulmonary tissues, with reductions in EPLIN transcript levels observed in higher grade, TNM stage and nodal involvement [30]. Similarly, Zhang et al. have further demonstrated, utilising both global databases and immunohistochemical staining, that EPLIN expression was reduced in metastatic prostate and colon tumours and in lymph node metastasis of prostate, colorectal, breast and squamous cell carcinoma of the head and neck (SCCHN) cancer [6]. Taken together these previous observations are largely consistent with our finding which implicated EPLIN as a protective factor in patients with gastric cancer.

The current study aimed to explore the relevance of EPLIN in relation to chemotherapy responsiveness. Surgical resection is the main treatment for gastric cancer. However, many gastric cancer patients are diagnosed at an advanced stage and surgery is not a suitable option. NAC is the administration of therapeutic agents used before surgery. Its aim is to shrink the size or extent of tumour for the purpose of possible removal, converting unresectable tumours into resectable tumours and/or decreasing micro metastatic disease. It is usually used for cancers which are too large to be removed easily and also can help indicate tumour sensitivity to chemotherapy [31]. NAC can decrease tumour TNM stage, increase surgical resection rate and decrease tumour related symptoms if successful [32]. NAC with docetaxel, oxaliplatin, fluorouracil or capecitabine are usually used in clinic and the application of NAC in gastric cancer therapy is important in order to improve survival rates [32].

Therefore, we further explored EPLIN expression in a smaller gastric cancer cohort containing information related to patient response to NAC. Interestingly, in the group with NAC, statistically lower EPLIN transcript expression was detected in tumour tissues than in normal tissues. While, in our first cohort, there was no significant difference in EPLIN expression between tumour tissues and normal tissues, though elevated levels were observed in normal tissues. This might indicate that EPLIN played a more important role in those larger tumours which required NAC. A lower EPLIN transcript level might lead to an increased tumour size which results in a requirement of NAC in order to shrink the tumour, so as to offer patients more surgical options.

Partially in keeping with this, lower EPLIN expression was seen in more aggressive tumours, such as late TNM stages, lymph node metastasis, deep tumour invasion to gastric wall, larger tumour size, and recurrence, though these lacked statistical significance. This indication is partially in line with the observations of breast and oesophageal cancer cohorts in our laboratory [7,8]. Survival analysis showed high EPLIN expression in gastric cancer patients with NAC led to a better OS, though it was not found to be significant in this smaller cohort, while EPLIN expression had little influence on DFS in patients with NAC.

Chemotherapy is a key therapy for gastric cancer, with NAC providing a possible treatment option for those with advanced disease. Nevertheless, one reason for poor survival of gastric cancer patients is due to chemotherapy resistance [33,34,35]. Currently, there is only limited data regarding the influence of EPLIN in responsiveness to chemotherapy. Zhang et al. demonstrated that EPLIN depletion significantly promoted cell chemoresistance to the treatment of docetaxel and doxorubicin in ARCaP_E_ prostate cancer cells [6]. In the current study, the relationship between EPLIN and gastric cancer chemosensitivity was investigated in patients with NAC. It was interesting to note that higher EPLIN expression was observed in the NAC responsive compared to NAC non-responsive tumours, though it did not reach significant impact in this relatively small cohort. We also carried out OS distributions to further explore the influence of EPLIN expression on responsiveness to NAC. This indicated that EPLIN is involved in chemotherapy sensitivity which contributed to overall survival. Taken together, EPLIN may negatively regulate the biological function of those gastric cancers which required NAC and might promote a long overall survival. Additionally, EPLIN might contribute to tumour responsiveness to chemotherapy, which might contribute to a better OS.

Together, the present study suggests that EPLIN might be a potential prognostic indicator of gastric cancer and play a role in responsiveness to chemotherapy. Given the high mortality rates attributed to metastasis and acquisition of therapy resistance, novel investigations into the associated mechanisms and regulatory pathways involved in such processes in gastric cancer are of key importance. Currently, EPLIN’s role in gastric cancer remains relatively uncharacterised, though it has been highlighted in other cancers as a metastasis suppressor with early implications in regulating therapy response. EPLIN therefore represents an interesting avenue for further investigation in gastric cancer and holds potential as a novel marker or prognostic indicator and also as a novel candidate potentially involved in therapy response. However, there are a number of limitations to our current study and much remains unknown. For example, our analysis was undertaken in relatively small cohorts, particularly so for the second cohort. Additionally, the observations reported result only from the expression analysis of clinical sample and lack laboratory investigation into gastric cancer cell model systems, both in vitro and in vivo. Finally, the mechanisms related to any impact of EPLIN on such aspects in gastric cancer also remain to be elucidated. Therefore, our initial findings require further studies and larger cohorts to gain a better understanding of the role of EPLIN in gastric cancer, especially in its involvement in chemoresistance and therapy response. Additional cell based and in vivo work, combined with mechanistic analysis is now required to fully assess the role of EPLIN in cell sensitivity to chemotherapeutics and to clarify its usefulness as a prognostic factor or indicator of therapy response in gastric cancer patients. Undertaking such work will provide novel opportunities for biomarker or therapeutic strategies related to EPLIN in gastric cancer.

## Figures and Tables

**Table 1:** Epithelial protein lost in neoplasm (EPLIN) log_10_ relative transcript expression in gastric cancer and normal tissues.

Characteristic	Sample Number (*n*)	Median Transcript Expression	Q1	Q3	*p* Value
Tissue Type					
Tumour	320	3.848	2.457	4.919	0.7288 ^a^
Normal	175	2.939	1.224	14.679	
Gender					0.4147 ^a^
Male	228	3.778	2.173	4.903	
Female	92	4.155	2.611	5.092	
T stage					0.121 ^b^
T1	16	4.21	2.653	4.97	
T2	26	4.589	3.114	5.357	
T3	41	3.445	1.855	4.622	
T4	229	3.847	2.07	4.911	
					0.0421 ^a^
T1 + T2	42	4.356	3.079	5.155	
T3 + T4	270	3.783	1.99	4.855	
Nodal involvement					0.859 ^b^
N0	70	4.154	2.059	5.054	
N1	48	3.836	1.9	5.068	
N2	65	3.847	2.44	5.005	
N3	131	3.786	2.666	4.775	
Metastasis status					0.9494 ^a^
M0	278	3.9	2.327	4.909	
M1	41	3.807	2.487	5.09	
TNM stage					0.596 ^b^
TNM1	25	4.352	2.983	5.047	
TNM2	60	3.977	1.735	5.245	
TNM3	217	3.786	2.487	4.798	
TNM4	9	5.09	0.35	5.46	
Borrmann classification					0.342 ^b^
BorrmannII	9	4.231	-1.122	4.58	
BorrmannIII	19	4.335	2.97	5.238	
BorrmannIV	7	1.29	-0.93	5.18	
					0.364 ^a^
BorrmannII + III	28	4.283	1.742	5.042	
BorrmannIV	7	1.291	-0.933	5.178	
Histopathologic type					0.45 ^b^
Adenocarcinoma	236	3.976	2.668	4.913	
Signet ring cell Carcinoma	5	4.235	1.768	5.146	
Mixed	47	3.359	1.884	4.775	
Differentiation					0.239 ^b^
High	1	−6.666	-	-	
High-Mid	6	4.555	4.03	5.525	
Mid	61	4.045	2.208	5.132	
Mid-Low	81	4.147	2.734	4.826	
Low	136	3.752	2.488	5.032	
Survival status					0.3168 ^a^
Alive	134	3.848	2.175	5.1	
Death	183	3.807	2.455	4.739	
					0.387 ^b^
Disease Free	119	3.847	2.666	5.131	
Metastasis	15	4.032	1.723	4.906	
Died of gastric cancer	183	3.807	2.455	4.739	

Q1: first quartile; Q3: third quartile; ^a^: Mann-Whitney U test; ^b^: Kruskal-Wallis test.

**Table 2:** EPLN log_10_ relative transcript expression in gastric cancer patients with neoadjuvant chemotherapy (NAC) and paratumour tissues.

Characteristic	Sample Number (*n*)	Median Transcript Expression	Q1	Q3	*p* Value
Tissue Type					
Tumour	78	1.135	0.325	1.816	<0.001 ^a^
Paratumour	80	2.73	2.255	3.921	
Gender					0.9867 ^a^
Male	56	1.118	0.32	1.827	
Female	22	1.16	0.334	1.816	
Borrmann classification					0.258 ^b^
BormannI	3	1.806	1.479	2.266	
BormannII	12	1.676	1.125	1.898	
BormannIII	31	1.162	0.15	1.923	
BormannIV	7	1.038	0.958	1.109	
					0.071 ^a^
BormannI + II	15	1.796	1.277	1.902	
BormannIII + IV	38	1.04	0.5404	1.818	
D2 Surgery status					0.9192 ^a^
No D2 Surgery	21	1.212	0.473	1.809	
D2 Surgery	57	1.109	0.152	1.83	
Diameter length					0.5512 ^a^
Diameter length > 50mm	36	0.976	0.152	1.74	
Diameter length < 50mm	32	1.298	0.386	1.801	
T stage					0.541 ^a^
T1 + T2	7	1.877	−0.605	1.902	
T3 + T4	70	1.092	0.325	1.796	
Nodal involvement					0.0979 ^a^
Node negative	12	1.707	1.195	1.946	
Node positive	66	1.028	0.277	1.8	
TNM stage					0.57 ^b^
TNM1	3	1.877	−0.605	2.266	
TNM2a	3	1.761	1.556	2.234	
TNM2b	8	1.196	0.082	1.632	
TNM3a	8	0.549	−0.505	1.793	
TNM3b	11	0.903	−0.053	1.797	
TNM3c	21	1.017	0.235	1.768	
TNM4	23	1.438	0.348	1.875	
					0.113 ^a^
TNM1 + 2a	6	1.819	1.016	2.242	
TNM2b,3,4	71	1.042	0.3169	1.797	
Recurrence status					0.269 ^a^
No recurrence	53	1.318	0.473	1.881	
Recurrence	24	0.93	0.064	1.781	
Survival status					0.3935 ^a^
Alive	26	1.334	0.451	1.879	
Death	52	1.04	0.198	1.801	
NAC response					0.3217 ^a^
Response to NAC	26	1.504	0.486	1.906	
No response to NAC	52	1.04	0.32	1.804	

Q1: first quartile; Q3: third quartile; ^a^: Mann-Whitney U test; ^b^: Kruskal-Wallis test.

**Table 3:** The implication of EPLIN transcript expression in clinical outcomes in patients with gastric cancer.

Analysis	Dependent Variable	OS	DFS
		*p* Value	
Univariate analysis	EPLIN	0.067	0.025
Multivariate analysis	TNM stage	<0.001	<0.001
	T stage	<0.001	<0.001
	Nodal involvement	<0.001	<0.001
	Metastasis	<0.001	<0.001
	Histology	0.188	0.268
	Invasion	<0.001	<0.001
	Embolism	<0.001	<0.001
	Differentiation	0.32	0.306
	EPLIN	0.024	0.015

## Data Availability

Data are available on reasonable request or were obtained through the kmplot website (www.kmplot.com).
